# Layout Dependence Stress Investigation in through Glass via Interposer Architecture Using a Submodeling Simulation Technique and a Factorial Design Approach

**DOI:** 10.3390/mi14081506

**Published:** 2023-07-27

**Authors:** Shih-Hung Wang, Wensyang Hsu, Yan-Yu Liou, Pei-Chen Huang, Chang-Chun Lee

**Affiliations:** 1 Department of Mechanical Engineering, National Yang Ming Chiao Tung University, No. 1001, Ta Hsueh Road, East District, Hsinchu City 300010, Taiwan; stockwang.en06@nycu.edu.tw; 2 Department of Power Mechanical Engineering, National Tsing Hua University, No. 101, Section 2, Kuang-Fu Road, Hsinchu City 30013, Taiwan; sq1356@livemail.tw (Y.-Y.L.); mars420225@gmail.com (P.-C.H.)

**Keywords:** multi-chiplet, TGV, finite element analysis, submodeling technique, analysis of variance

## Abstract

The multi-chiplet technique is expected to be a promising solution to achieve high-density system integration with low power consumption and high usage ratio. This technique can be integrated with a glass interposer to accomplish a competitive low fabrication cost compared with the silicon-based interposer architecture. In this study, process-oriented stress simulation is performed by the element activation and deactivation technique in finite element analysis architecture. The submodeling technique is also utilized to mostly conquer the scale mismatch and difficulty in mesh gridding design. It is also used to analyze the thermomechanical responses of glass interposers with chiplet arrangements and capped epoxy molding compounds (EMC) during curing. A three-factor, three-level full factorial design is applied using the analysis of variance method to explore the significance of various structural design parameters for stress generation. Analytic results reveal that the maximum first principal stresses of 130.75 and 17.18 MPa are introduced on the sidewall of Cu-filled via and the bottom of the glass interposer, respectively. Moreover, the EMC thickness and through glass via pitch are the dominant factors in the adopted vehicle. They significantly influence the stress magnitude during heating and cooling.

## 1. Introduction

The concept of three-dimensional integration of advanced electronic packaging has been widely developed and adopted in the semiconductor industries, and corresponding solutions for interconnect systems are becoming a key technique. From the viewpoint of interconnect systems, a vertical stacked structure is considered a promising solution to achieve integration density and multiple functionalities. Accordingly, the interposer architecture is demonstrated with silicon (Si) and a glass material system for the performance requirement of ultrafine pitch features. Glass-based interposers have been regarded as a superior alternative to Si-based interposer architecture because of their thermal and electrical properties and low cost [1,2]. Glass interposers are targeted to reduce the cost of high-density integration and compatibility with the large pan size (450–700 mm) procedure [1]. However, the problem of the low yield of glass interposers with through glass via (TGV) remains unresolved, especially with regards to the cracking behavior and electrical degradation under thermal cycling loading [2]. The adjustable coefficient of thermal expansion (CTE) is an effective solution to manage the thermomechanical responses of glass interposer structures, and a combination of a thin sputtered layer with an electroless plating metal layer was demonstrated as a viable method to form TGV with high aspect ratios [3]. Among all the possible material systems of interposers, the overall characteristics of glass are better than those of other materials to accomplish more frequency bands, smaller form factors, and lower power consumption [4]. A handling procedure of glass wafers on Si handles was demonstrated with a polymer-free temporary bonding process [5]; the present handle approach was validated at 400 °C without outgassing and significantly improved the reliability during the handle. The helium hermeticity reliability of copper (Cu)-filled TGV wafers was tested under different harsh environmental conditions, including thermal shock, high-temperature storage, and highly accelerated temperature and humidity stress tests [6]. Thermomechanical reliability issues of glass interposers were investigated, and several glass interposers with different material characteristics were adopted to minimize the thermomechanical responses of temperature-related processes [7,8]. Laser-induced deep etching technology was used to achieve high aspect ratio features (close to 1:100) and prevent significant internal and superficial defects [9]. To address the aforementioned reliability issues, finite element analysis (FEA) simulation was utilized to estimate the stress and warpage effect on interposers generated from annealing and single- and double-side processing [10].

In addition, the failure mechanism and optimization rule of TGV interposer architecture were systemically investigated [11,12,13,14,15,16,17,18,19]. Tensile radial and circumferential stresses were attributed to the origin of the circumferential cracks and the formation of radial cracks, respectively [11,13,14]. Different annealing procedures were designed to study their influence on Cu protrusion mechanisms [12,15], and Cu protrusion was observed to saturate after a dwell time of 4 h with an annealing temperature of 400 °C. Irreversible Cu protrusion of Cu-filled TGV was generated after thermal cycling loading and is attributed to the plastic deformation and creep mechanism of Cu [16]. Different layout designs, such as fully filled via and conformal via, were demonstrated and investigated in terms of their thermomechanical reliability [17]. Interfacial delamination between glass interposers and Cu-filled TGV was explored, and the corresponding energy release rate was proportional to the via diameter and the thermal mismatch strain, which was highly dependent on the layout design parameters [18]. A metallization process filled with Ag-paste composite solution was presented, and its effectiveness was compared with that of the general Cu electroplating process; the analytical results revealed that the aforementioned metallization process would introduce different cracking behaviors [19]. The analytical model and FEA simulation approach have been widely adopted to reduce the time-consuming experimental work due to the cost of the semiconductor fabrication process [20,21,22]. An analytical model was derived to estimate the thermal stress and warpage in terms of different geometrical parameters and material selections [20,21]. Heating temperature, dwell time, gap width, and surface tension were interpolated in the derived analytical model, and they influenced the reflow speed [22]. The glass reflow mechanism and corresponding thermomechanical stress generated in a glass–Si composite interposer were explored [23]. Moreover, specific shrinkage phenomena in epoxy molding compound (EMC) and substrate material during the assembly reflow process were demonstrated and validated by warpage profile comparison [24]. The selection of EMC material significantly influences the packaging warpage and solder joint fatigue life [25,26].

From the viewpoint of electron packaging applications, the design concept of chiplet arrangement was proposed to improve the yield and reduce product costs [27,28]. A dual-chiplet, interposer-based system-in-package architecture was demonstrated to establish a high-performance computing processor design, and the data rate of up to 8 Gb/s with relatively low power and area overhead was explored [29]. The development and manufacturing cost of AMD’s 32-core CPU was reduced by 40% because of the chiplet design; this performance revealed its advantage in cost reduction [30]. Cost and yield tradeoff of chiplet and monolithic chip integration were analyzed with possibly uncertain parameters; the results show that the overall cost of chiplet design is lower than that of the monolithic chip in five-year business planning [31]. The integrated fan-out (FO) on substrate solution was demonstrated by TSMC to achieve advanced chiplet integration; the mechanical reliability and fatigue risk of the present vehicle under temperature and humidity tests were assessed [32,33,34].

In the chiplet integration design, the microbump, through via, and redistribution layer (RDL) are still regarded as the major interconnection components in 2.5D integration technology [35]. Multilayer RDL interposer packaging is regarded as a promising solution for heterogeneous integration platforms; six-layer interconnection is provided for design flexibility of chiplets and high bandwidth integration in this solution [36]. In view of the thermomechanical concerns, chiplet arrangement design is harmful to the stability of electron packaging architecture because of the lower stiffness of separated chiplets than that of the single chip. Moreover, the spacing between chiplets is filled by EMC, and the overall deformation of electronic packaging vehicles is aggravated due to CTE mismatch and EMC chemical shrinkage. Chip-last, process-based FO multi-chiplet integration design was developed, and its process-induced warpage and RDL stress issues were analyzed [37]. The ring- and cavity-type heat spreaders are designed to improve the warpage behavior of the concerned vehicle by the high stiffness of the heat spreader. A design concept of glass panel embedding technology was proposed; it embeds the concerned chip in the glass substrates with plated RDL and TGV to achieve a trace below 2 μm by adopting polymer RDL, providing a solution for warpage control [38]. Cu bridge design improves upon conformal Cu-filled via structures and has superior reliability against thermal stress [39].

**Figure 1:**
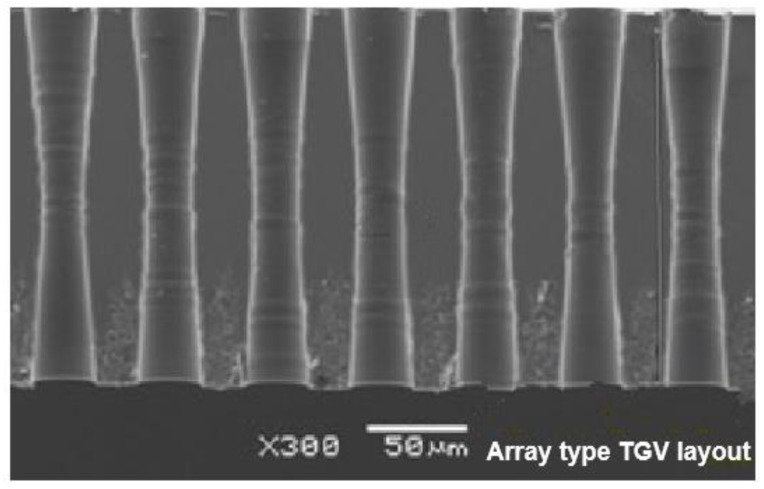
Cross-sectional view of array-type TGV layout in glass interposer architecture.

The stress-induced failure in chiplet applications is costly and unbearable because the failure of a single die would cause failure of the monolithic chip [30], but current studies are focused on the warpage behavior investigation of multi-chiplet systems [32,37]. Accordingly, the stress generation mechanism during fabrication of chiplet integration vehicles needs to be explored, and the induced stress needs to be managed to improve long-term reliability. In this study, the FEA-based submodeling technique integrated with the equivalent material approach is used to simulate the process-oriented thermomechanical responses of glass interposer architecture integrated with multi-chiplet design; problems related to prediction accuracy and computing resources of the glass interposer model are resolved with a fine-pitch TGV array (Figure 1). The process-oriented simulation is enabled by the element activation and deactivation technique and is used to explore the stress generation mechanism of TGV interposers with chiplet arrangement during curing. Moreover, the analysis of variance (ANOVA) is utilized to reveal the significance of different structural layout design parameters (namely TGV via diameters, TGV pitch, and EMC thickness) on stress generation in the glass interposer in question.

## 2. Structural Layout Design and Fabrication Process of the Cu-Filled TGV Interposer

**Figure 2:**
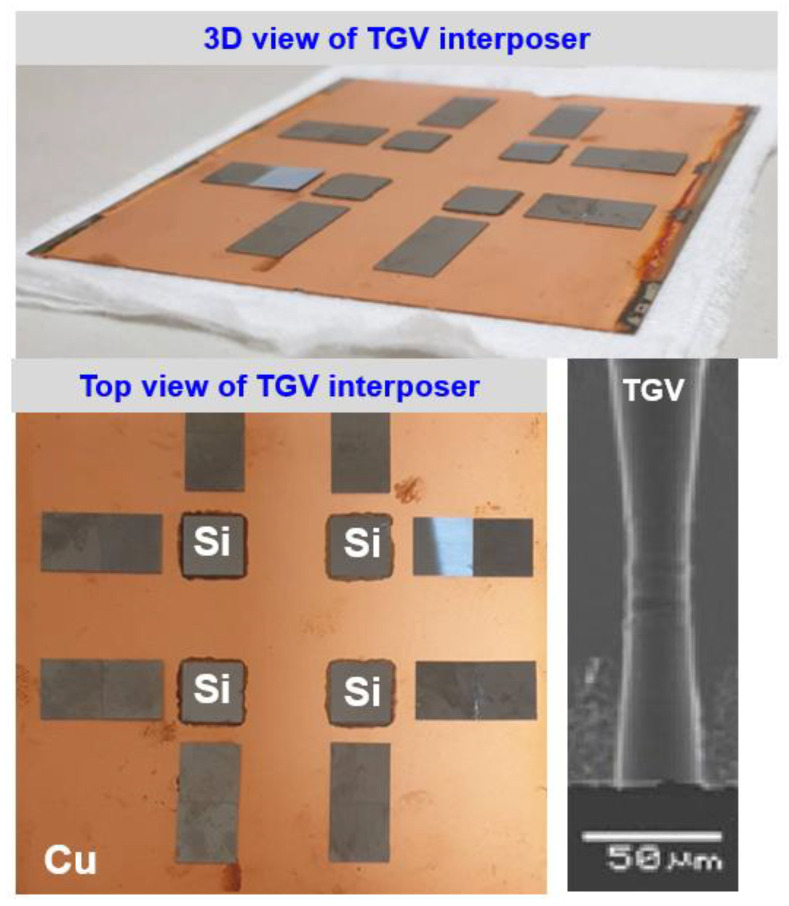
Optical and SEM image of the utilized glass interposer vehicle with multi-chiplet arrangement design.

As illustrated in Figure 2, the utilized glass interposer vehicle has a 100 mm × 100 mm area and eight chips, including four Si chips with a 10 mm × 10 mm area and four Si chips with a 10 mm × 20 mm area. The gap between each chip is 15 mm. The thicknesses of the Si chip, glass interposer, and EMC are designed as 0.2, 0.5, and 0.5 mm, respectively. The considered structural layout parameters of TGV, namely via diameter and via pitch, are defined as 28 μm and 1 mm, respectively, for the baseline design. The detailed fabrication process was demonstrated in the previous study [40]. The process steps are described as follows. Eight Si chips are mounted on the Cu metallization layer surface at room temperature. The glass interposer is fixed at the vacuum platform to prevent sliding during subsequent curing and cooling. The chamber is preheated up to 130 °C, which is designed as the curing process temperature. A compressive loading of 5.5 kgf/cm^2^ (0.539 MPa) is subsequently loaded to complete the curing process of the utilized EMC. The loaded compression load is removed, and the glass interposer vehicle is cooled down to room temperature to complete the entire fabrication process. Notably, the specific material characteristic of the adopted EMC, namely chemical shrinkage, is 0.1083% under fully cured conditions.

## 3. Utilizing the Equivalent Material Approach and Submodeling Technique for the Stress Distribution Estimation of the TGV Interposer with Multi-Chiplet Arrangement

### 3.1. Extraction Approach of the Equivalent Mechanical Property for the TGV Interposer

**Figure 3:**
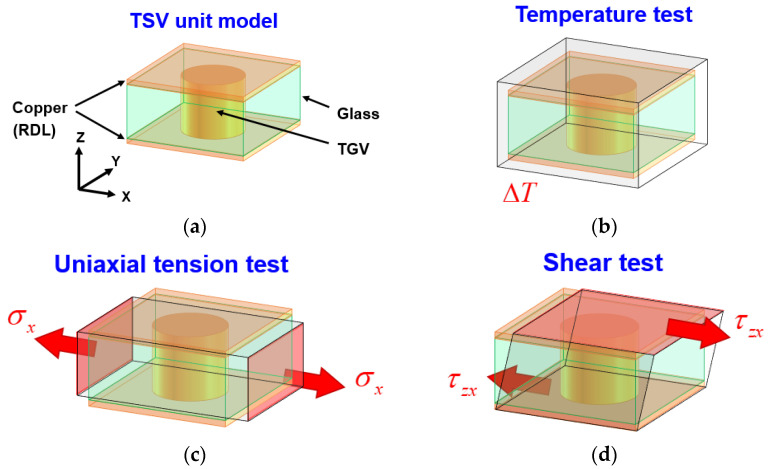
Graphical explanation of equivalent material property extraction on the glass interposer: (**a**) representative TGV unit cell; (**b**) schematic of temperature test; (**c**) schematic of uniaxial tension test; (**d**) schematic of shear test.

The equivalent material test approach is utilized in this study to resolve the mesh gridding and numerical convergence issue of FEA modeling with over thousands of Cu-filled TGV. Three types of material tests, namely temperature change, uniaxial tension, and shear tests, are performed to extract the corresponding CTE, Young’s modulus, and shear modulus of a single unit of the TGV array, as illustrated in Figure 3. As shown in Figure 3a, the single-unit TGV is composed of a glass interposer, Cu-filled via, and a Cu RDL layer. This TGV unit can be regarded as the representative volume element in the entire TGV array and is used to simplify the difficulty in FEA modeling construction by utilizing the equivalent material test approach. The material properties utilized in the present simulation work are listed in Table 1. The material characteristics of glass and Cu are adopted into the extraction of equivalent material properties of TGV unit cells. The CTE behavior is the critical property to estimate the thermomechanical response of the present vehicle. It can be extracted by the deformation of TGV unit cells under a given temperature change Δ*T* (Figure 3b). The corresponding strain along each axis is expressed as follows:

(1) $$\varepsilon_{x}=\alpha_{x}\Delta T,\;\varepsilon_{y}=\alpha_{y}\Delta T,\;\varepsilon_{z}=\alpha_{z}\Delta T$$

where *ε* denotes the induced strain under a given temperature change and *α* is the CTE characteristic.

The equivalent Young’s modulus along each axis can be extracted in accordance with the ratio relationship of applied normal stress divided by the corresponding induced strain (Figure 3c). Notably, all the displacement degrees of freedom (DOFs) on the central point of TGV unit cells are fixed to prevent the rigid body motion during the uniaxial tension test. The equivalent Young’s modulus is separately extracted by the tension test along different axes, and the equivalent Poisson’s ratio can also be calculated by the ratio of strain amplitude between any two orthogonal axes. The detailed expressions of equivalent Young’s modulus and Poisson’s ratio estimation are

(2) $$\sigma_{x}=E_{eq\_x}\varepsilon_{x},\;\sigma_{y}=E_{eq\_y}\varepsilon_{y},\;\sigma_{z}=E_{eq\_z}\varepsilon_{z}$$

(3) νeq_xy=−εyεx, νeq_xz=−εzεx, νeq_yz=−εyεz

where *σ*, *E*, and *ν* represent the applied normal stress, Young’s modulus, and Poisson’s ratio, respectively.

The equivalent shear test is performed to extract the corresponding shear modulus of the TGV unit cell. As illustrated in Figure 3d, the displacement DOF on the bottom surface of the TGV unit cell is fixed. An external constant displacement is subsequently applied to the opposite surface. Under the assumption of a small deformation, the shear strain is determined by the ratio of a given displacement/height of the TGV unit cell. Moreover, the reacting force on the bottom surface of the TGV unit cell is induced, while the foregoing constant displacement is exerted. Accordingly, the equivalent shear modulus can be calculated by

(4) $$\tau_{xz}=G_{eq\_xz}\gamma_{xz},\;\tau_{xy}=G_{eq\_xy}\gamma_{xy},\;\tau_{yz}=G_{eq\_yz}\gamma_{yz}$$

where *τ*, *G*, and *γ* denote the shear stress, shear modulus, and shear strain in the shear test, respectively. The structural dimension dependence of the TGV unit cell can be effectively estimated using the aforementioned equivalent material tests. The corresponding equivalent material properties under different TGV diameters and pitches are extracted and summarized in Table 2. These properties are utilized in the following FEA simulation work with various TGV layout parameters.

### 3.2. Methodology Validation of the Equivalent Mechanical Property and TGV FEA Model

**Figure 4:**
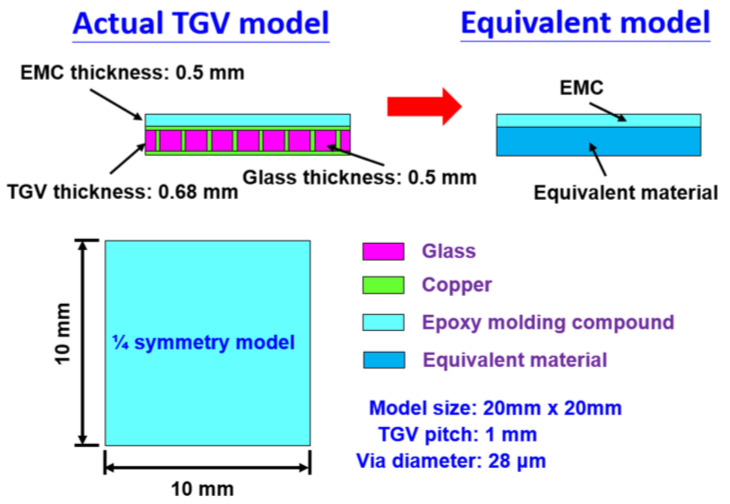
The schematic diagram of geometrical configuration and material composition for the demonstration of the present simulation methodology by using the TGV FEA model with equivalent material layer and actual TGV framework, respectively.

**Figure 5:**
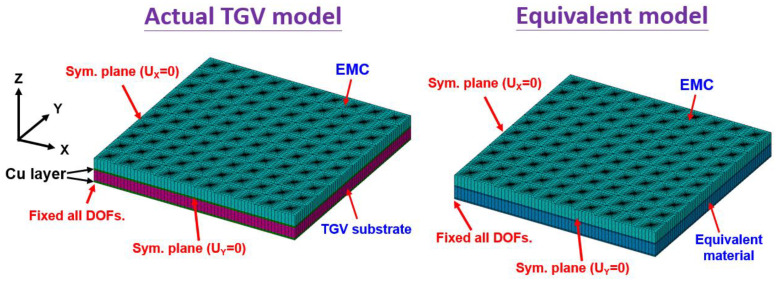
The finite element models of actual and equivalent TGV interposers with multi-chiplet arrangements.

**Figure 6:**
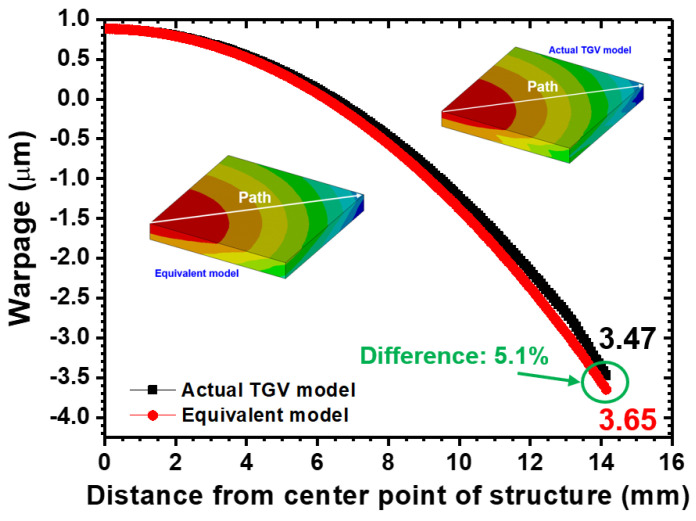
A warpage profile comparison of actual and equivalent TGV FEA models along the diagonal path when a raised temperature loading from 25 °C to 130 °C is given.

In order to validate that the equivalent mechanical properties estimated in the proposed FEA model are highly reliable, a comparison of FEA models is performed between an actual Cu-filled TGV interposer and an equivalent architecture by considering a smaller structural scale of 20 mm × 20 mm. In the schematic diagram cross-sectional view shown in Figure 4, a 0.5 mm thickness of EMC is located on the TGV interposer composed of a glass substance and array-type Cu-filled vias measuring 28 μm in diameter. It is noticed that a covered Cu layer is separately at the top and bottom sides of the foregoing interposer to suppose them as the interconnect patterns. Meanwhile, the chips are not included in simulation models to ignore the effect of multi-chiplet arrangement on structural deformation. After implementing the equivalent material of the TGV interposer in accordance with the approaches introduced in the aforementioned section, the concerned TGV structure of the FEA model is simplified to a bi-layered configuration composed of EMC and an equivalent layer. In the demonstration of simulation methodology, a quarter symmetry model is taken into account due to the whole interposer having the biaxial symmetrical characteristic. The corresponding boundary constraints applied to both FEA models are respectively shown in Figure 5. Based on the geometrical conditions and the percentage of Cu-filled vias, the estimated equivalent mechanical properties (diameter = 28 μm, pitch = 1 mm) listed in Table 2 are utilized in the simulations. The analytic results of structural warpage by applying a temperature loading from 25 °C to 130 °C are shown in Figure 6. The analytic results indicate that the maximum warp occurs on the corner furthest from the structural center. Only a small difference of 5.1% in the estimated warpage magnitude is acquired, as compared to the actual TGV model and equivalent model. In other words, the thermal deformation obtained from the equivalent FEA model is almost identical to the results estimated from the actual model. Therefore, the proposed simulation methodology with equivalent material approach is validated to have the capability of accurately maintaining the mechanical behavior of the original detailed construction of the interposer model. Based on this validation, the following analyses and discussion are consequently performed.

### 3.3. FEA Modeling of the TGV Interposer with Multi-Chiplet Arrangement

**Figure 7:**
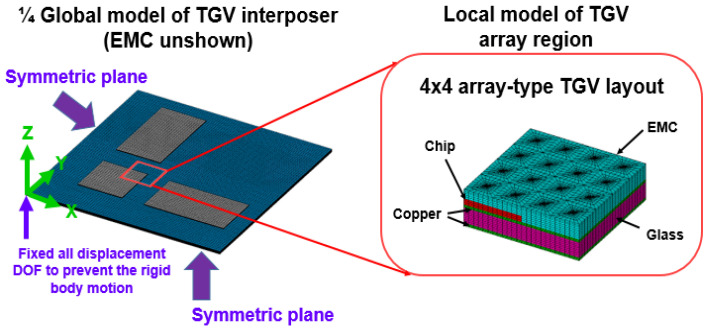
Fabrication process diagram of the glass interposer with array-type and process conditions and material components.

The glass interposer architecture utilized in this study comprises a 100 mm ×100 mm interposer and array-type Cu-filled TGV with a via diameter of 28 μm and a TGV pitch of 1 mm. Multi-chiplet arrangement design is further attached on the glass interposer, unlike the original vehicle utilized in the previous study [40]. As shown in Figure 7, a one-quarter FEA model of an entire glass interposer vehicle is built in accordance with the symmetry of structure design. The boundary condition of the constructed model is described as follows. The inner side planes are set as symmetric planes based on the symmetry of the utilized glass interposer vehicle. All the displacement DOFs on the bottom of the central axis are fixed to prevent movement of the rigid body during the mechanical response simulation. The concerned region is regarded as the local model and constructed with detailed components: Si chip, glass interposer, Cu metallization layer, and molded EMC. In the simulation work, the submodeling technique is utilized and integrated with an equivalent material approach to further overcome the difficulty in mesh gridding and convergence issues. The displacement field generated from the thermocompression process of the global glass interposer model is extracted and subsequently interpolated into the local model as the boundary conditions. The thermocompression process-induced mechanical responses are introduced in the concerned region. The accuracy and stability of the prediction are also validated, which indicates that at least a 4 × 4 TGV array needs to be constructed in the local model to gain a stable and reliable simulation result.

### 3.4. Stress Distribution and Generation Mechanism of the TGV Interposer with Multi-Chiplet Arrangement under the Thermocompression Process Loading

**Figure 8:**
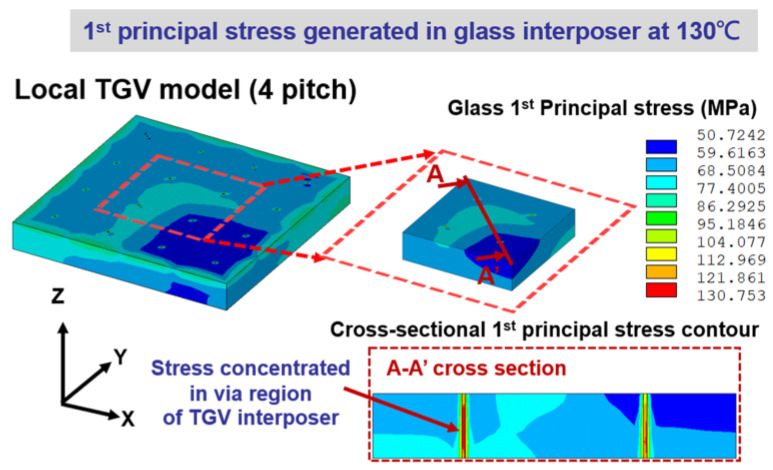
Simulated first principal stress contour of the 4 × 4 TGV array region under the temperature loading of 130 °C during curing.

To explore the critical stress location in curing, the simulated stress contour of the glass interposer with a Cu-filled TGV array at the curing temperature of 130 °C is shown in Figure 8. A maximum first principal stress of 130.75 MPa is observed, and it is not generated on the external surface of the TGV interposer but is detected in the TGV array region. A detailed stress contour of the TGV array region is extracted, and the cross-sectional stress contour through the A–A’ line reveals that the curing process-induced stress is concentrated at the sidewall region of TGV via. This stress generation mechanism can be explained by the CTE mismatch phenomenon between major components in the present vehicle, namely Cu, glass interposer, and capped EMC. From the viewpoint of solid mechanics, the stress in the temperature-related process is generated by the CTE mismatch and the corresponding thermal deformation difference. The CTE characteristics of the three aforementioned components are 18, 0.52, and 8.5 ppm/K, respectively. Under the temperature loading, the CTE mismatch between Cu/glass and glass/EMC are estimated to be different by 34.61 and 16.35 times. Accordingly, larger CTE mismatch-induced deformation is generated at the Cu/glass interface than at the glass/EMC interface. Potential cracking might occur in the glass interposer or the bonded interface between the glass interposer and Cu-filled via. These phenomena are also explored in Okoro’s work [13,14]. The aforementioned literature mentions that the radial cohesive crack might form in the glass during heating, and the Cu metallization thickness dependence stress generated in glass substrate is estimated. The maximum stress of the glass interposer under 130 °C temperature loading presented in the current study is comparable to the results revealed in Okoro’s work [13]. However, the other TGV-related structural layout designs, including TGV via diameter and TGV pitch, are not discussed in the literature. A parametric study considering the aforementioned structural layout parameters is performed, and the significance of each parameter is analyzed by ANOVA to explore the influences of foregoing layout parameters on stress generation in the TGV interposer.

**Figure 9:**
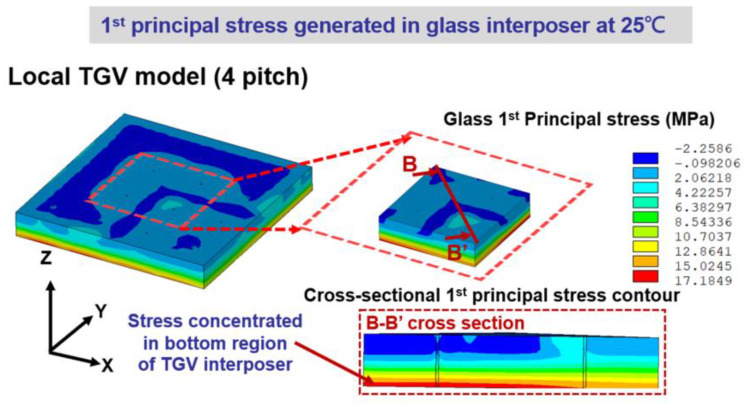
Simulated first principal stress contour of the 4 × 4 TGV array region after the TGV interposer vehicle cools down to the room temperature of 25 °C.

Stress introduced during cooling might cause the circumferential-type cracking in TGV architecture [13,14]. For this reason, the stress contour of the glass interposer structure is also extracted to investigate the stress distribution and magnitude generated by the cooling procedure. As shown in Figure 9, the critical stress location is revealed as the bottom plane of the TGV interposer. This phenomenon is attributed to the chemical shrinkage deformation of EMC generated by the curing procedure. For the EMC component utilized in this study, the chemical shrinkage amount is considered to be 0.1083% under fully cured conditions. The foregoing shrinkage deformation compresses the chip and Cu metallization layer and generates a concave-type bending profile on the glass interposer. Accordingly, the stress distribution of the TGV interposer is similar to the general bending stress distribution, and the maximum first principal stress is introduced on the bottom plane of the glass substrate. This shrinkage deformation dominates the stress distribution of the entire vehicle rather than the CTE mismatch mechanism between each component, which is due to the CTE mismatch during room temperature being limited. The chemical shrinkage-induced stress in the TGV interposer architecture after cooling is significantly lower than the stress level generated from the curing procedure. However, the concave bending profile might cause the misalignment issue and affect the stability of the concerned vehicle in subsequent assembly or reliability tests. Accordingly, a parametric study is also performed to estimate the stress generated in the TGV interposer in question after cooling.

### 3.5. Sensitivity Analysis of Structural Parameters on Stress Generation of the TGV Interposer

**Figure 10:**
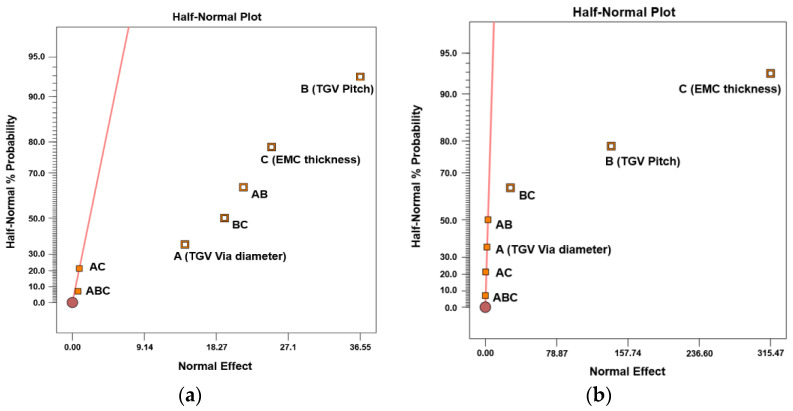
Half-normal probability plot of stress magnitude generated in TGV interposer architecture: (**a**) during curing at 130 °C; (**b**) after cooling down to room temperature.

A three-factor, three-level full factorial design based on ANOVA is used to explore the significance of several layout design parameters for stress generation. In this analysis, structural design factors, namely TGV via diameter, TGV pitch, and EMC thickness, are utilized to check their effect on the induced first principal stress of the glass interposer. The low, medium, and high levels of TGV via diameter and pitch are considered to be 28, 44, and 60 μm and 0.5, 1, and 2 mm, respectively. The three levels of designed EMC thickness are 250, 375, and 500 μm, respectively. The half-normal probability plots of first principal stress generation during curing at 130 °C and the subsequent cooling process are illustrated in Figure 10a,b. In Figure 10, the factor mark away from the fitting line represents its significance on the first principal stress generation. In Figure 10a, the induced stress during curing is mainly dominated by the design factor B (TGV pitch), followed by the designed EMC thickness. The interaction effect between designed factors, namely AB (TGV diameter and pitch) and BC (TGV pitch and EMC thickness), slightly influences the first principal stress introduced in the glass interposer. Similar to the trend revealed in Figure 10a, the stress-induced significance from cooling is also illustrated in Figure 10b. It is dominated by the EMC thickness, followed by the TGV pitch. However, the interaction effect between design factors is nearly negligible. This phenomenon is attributed to the shrinkage deformation amount depending on EMC thickness, and the TGV pitch is the major factor that affects the overall stiffness of the Cu-filled TGV interposer because of the higher stiffness of Cu than that of glass. A detailed discussion on the stress generation mechanism and related design rule exploration is described in the following section.

## 4. Results and Discussion

### 4.1. Stress Estimation of the Glass Interposer during High-Temperature Curing with Various Structural Layout Design Parameters

**Figure 11:**
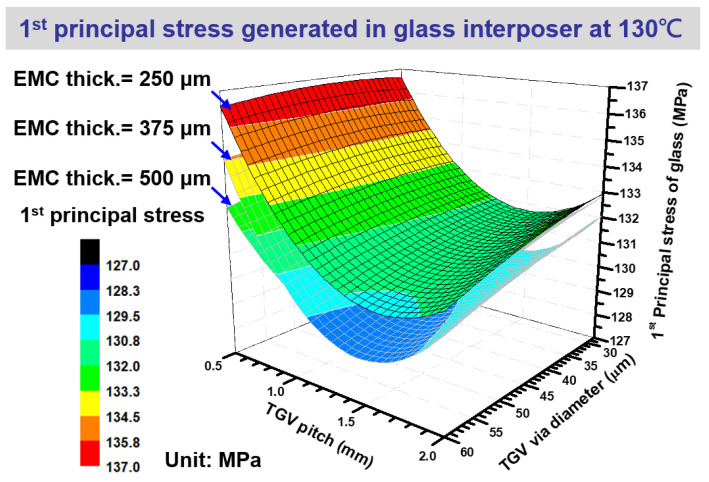
Comprehensive first principal stress influence on the glass interposer with various EMC thicknesses, TGV via diameters, and TGV pitches during curing at 130 °C.

The layout dependence first principal stress generated in the designed TGV interposer during curing at 130 °C is illustrated in Figure 11. Multiple stress trends on the glass interposer can be observed under the integrated influence of EMC thickness, TGV diameter, and TGV pitch design. The introduced first principal stress is proportional to the decrease in EMC thickness. The curing process-induced stress on the glass interposer is increased from 130.8 MPa to 132.6 MPa when the EMC thickness is decreased from 500 μm to 250 μm. Notably, the TGV diameter and pitch are fixed at the baseline designs of 28 μm and 1 mm, respectively. The aforementioned phenomenon can be attributed to the thinned EMC thickness decreasing the rigidity of the EMC, and the foregoing EMC will be constrained by the Cu layer above the glass interposer. In other words, the Cu layer can be regarded as a buffer layer to prevent the extrusion of glass under the thermal expansion of EMC at 130 °C. Moreover, the stress influence of varied EMC thickness is considered to insignificantly cooperate with other structural parameters, namely TGV via diameter and TGV pitch. Therefore, the interactive effect on stress generation in TGV interposer architecture is mainly induced on the structural characteristics of TGV via, including via diameter and pitch design. The interactive effect of different TGV via diameters and pitch designs on the first principal stress of the glass interposer is also expressed in Figure 11. The generated first principal stress is relatively limited in the TGV pitch design of 1 μm, but higher stress values are observed at the pitch designs of 0.5 and 2 μm. The phenomenon might be explained by the rigidity change of the TGV array in glass interposer architecture. The TGV array with a pitch design of 1 μm has a well-balanced rigidity that can handle the chemical shrinkage of EMC. It also has a decent flexibility to release partial stress through deformation. The TGV array with a via pitch design of 0.5 μm has difficulty releasing the stress through its flexibility because the narrow via pitch of the TGV array enhances its stiffness, and the TGV array with a via pitch design of 2 μm cannot separate the curing process-induced stress to multiple TGV via because of the large via pitch between each TGV via. Accordingly, the different EMC thickness design is regarded as manageable because of its linear influence on the generated first principal stress of glass. However, the design of TGV via diameter and pitch should be explored carefully while considering the interactive effect between foregoing structural parameters.

### 4.2. Stress Estimation on the Glass Interposer during Cooling with Various Structural Layout Design Parameters

**Figure 12:**
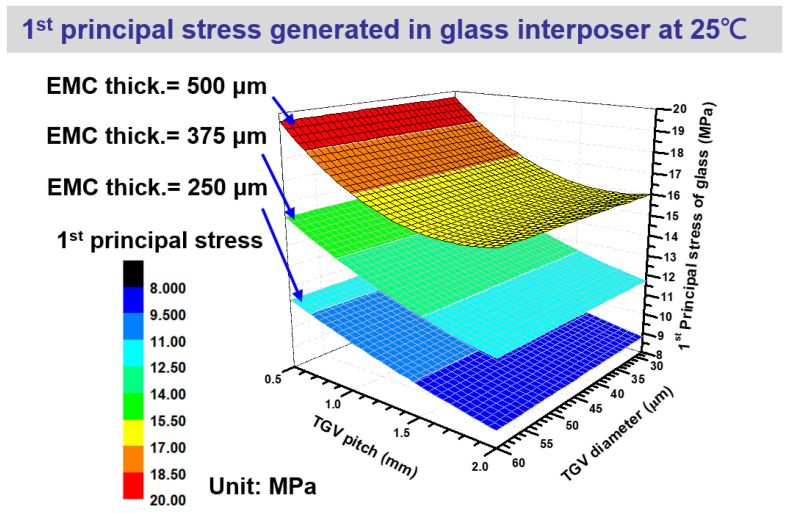
Comprehensive first principal stress influence of the glass interposer with various EMC thicknesses, TGV via diameters, and TGV pitches after curing and cooling down to room temperature.

After the curing process of the EMC at 130 °C, the entire glass interposer is cooled down to the room temperature (25 °C in the present fabrication process). Moreover, the critical stress location is transferred from the sidewall of TGV via to the bottom of the glass interposer. As illustrated in Figure 12, the first principal stress introduced in the glass interposer is dominated by the designed EMC thickness, and it is slightly influenced by the TGV pitch. Notably, the TGV diameter is observed with approximately zero effect on the induced first principal stress. Based on the stress generation mechanism revealed in Figure 9, the chemical shrinkage of EMC dominates the stress magnitude and distribution of the glass interposer, which is cooled down to room temperature. Accordingly, the stress is enhanced from 11.47 MPa to 19.47 MPa when the EMC thickness is increased from 250 μm to 500 μm, with an increased volume of EMC and an enlarged corresponding chemical shrinkage amount. Moreover, the increased TGV pitch suppresses the concentrated stress on the bottom plane of the glass interposer. This phenomenon is attributed to the enlarged TGV pitch also decreasing the density of the TGV array and the corresponding stiffness. Accordingly, the glass interposer can release the suffered stress by the flexibility of the glass interposer itself. From the aforementioned discussion, the stress generation mechanism of the glass interposer with multi-chiplet integration under thermocompression and curing process is investigated. The critical stress locations during heating and cooling are the sidewall of Cu-filled via and the bottom surface of the glass interposer, respectively. The maximum simulated first principal stresses of the glass interposer during curing and cooling are 132.60 and 19.47 MPa, respectively. The process-induced stress of the glass interposer after cooling is relatively manageable compared with the stress introduced from the curing process. Accordingly, the critical process step in the present glass interposer architecture with multi-chiplet arrangement is explored as the curing procedure. The corresponding process parameters will be an important issue to be addressed for subsequently improving the mechanical reliability of the glass interposer structure. The arrangement design of multi-chiplet integration will also be a notable factor when the dense and specific chiplet arrangement with different overall stiffness and volume proportions of capped EMC is considered in the future.

## 5. Conclusions

In this study, the comprehensive stress influence of the glass interposer integrated with multi-chiplet arrangement is investigated by the FEA-based process-oriented stress simulation and submodeling technique. The curing process is explored as a critical process with high potential cracking risk because the harsh stress is observed on the sidewall of the Cu-filled via region of the glass interposer with multi-chiplet arrangement. A three-factor, three-level full factorial design analysis is performed based on ANOVA to explore the dominant design factor of process-induced stress generation. The design factors of TGV pitches and EMC thicknesses are the dominant factors on introduced stress magnitude during curing and cooling, respectively, and the interaction effect between each design factor is insignificant. Notably, a decent trade-off on the designed EMC thickness is needed to balance the induced stress magnitude in the fabrication process of TGV interposer architecture. From the stress induced by the curing process with a temperature loading of 130 °C, the introduced first principal stress is up to approximately 130 MPa and might cause the brittle cracking phenomenon on the glass interposer itself. This study contributes to the literature by exploring the design rules for thermomechanical stress management of glass interposer architecture with Cu-filled TGV structure of chiplet arrangement design.

## Figures and Tables

**Table 1:** Material properties utilized in the FEA stress simulation of the present glass interposer with chiplet arrangement.

Components	E (GPa)	ν	CTE (ppm/K)
EMC	8	0.30	8.5
Si chip	169	0.30	3
Interposer (Corning HPFS^®^ glass, (Corning, NY, USA))	73	0.16	0.52
Cu	115	0.34	18

**Table 2:** Structural dimension dependence of the TGV unit cell under different TGV diameters and pitches.

TGV Parameters			E (GPa)		G (GPa)		Poisson’s Ratio		CTE (ppm/K)
Diameter	60 µm	X	85.4389	XY	34.7356	XY	0.230	X	7.96
Pitch	0.5 mm	Y	85.4389	YZ	34.7336	YZ	0.215	Y	7.96
		Z	81.9116	XZ	34.7336	XZ	0.215	Z	6.12
Diameter	44 µm	X	85.3156	XY	34.6976	XY	0.230	X	7.90
Pitch	0.5 mm	Y	85.3156	YZ	34.6964	YZ	0.214	Y	7.90
		Z	81.7130	XZ	34.6964	XZ	0.214	Z	6.01
Diameter	28 µm	X	85.2308	XY	34.6713	XY	0.229	X	7.85
Pitch	0.5 mm	Y	85.2308	YZ	34.6708	YZ	0.214	Y	7.85
		Z	81.5743	XZ	34.6708	XZ	0.214	Z	5.93
Diameter	60 µm	X	85.2397	XY	34.6740	XY	0.229	X	7.85
Pitch	1 mm	Y	85.2397	YZ	34.6735	YZ	0.214	Y	7.85
		Z	81.5870	XZ	34.6735	XZ	0.214	Z	5.94
Diameter	44 µm	X	85.2090	XY	34.6645	XY	0.229	X	7.84
Pitch	1 mm	Y	85.2090	YZ	34.6643	YZ	0.213	Y	7.84
		Z	81.5374	XZ	34.6643	XZ	0.213	Z	5.91
Diameter	28 µm	X	85.1878	XY	34.6580	XY	0.229	X	7.83
Pitch	1 mm	Y	85.1878	YZ	34.6579	YZ	0.213	Y	7.83
		Z	81.5027	XZ	34.6579	XZ	0.213	Z	5.89
Diameter	60 µm	X	85.1900	XY	34.6587	XY	0.229	X	7.83
Pitch	2 mm	Y	85.1900	YZ	34.6585	YZ	0.213	Y	7.83
		Z	81.8059	XZ	34.6585	XZ	0.213	Z	5.89
Diameter	44 µm	X	85.1823	XY	34.6563	XY	0.229	X	7.82
Pitch	2 mm	Y	85.1823	YZ	34.6562	YZ	0.213	Y	7.82
		Z	81.4934	XZ	34.6562	XZ	0.213	Z	5.89
Diameter	28 µm	X	85.1770	XY	34.6546	XY	0.229	X	7.82
Pitch	2 mm	Y	85.1770	YZ	34.6546	YZ	0.213	Y	7.82
		Z	81.4848	XZ	34.6546	XZ	0.213	Z	5.88

## Data Availability

Not applicable.
